# Climate change adaptation in South Africa: a case study on the role of the health sector

**DOI:** 10.1186/s12992-019-0466-x

**Published:** 2019-03-19

**Authors:** Matthew F. Chersich, Caradee Y. Wright

**Affiliations:** 1 Wits Reproductive Health and HIV Institute, Faculty of Health Sciences, University of the Witwatersrand, Johannesburg, South Africa; 2 Environment and Health Research Unit, South African Medical Research Council and Department of Geography, Geoinformatics and Meteorology, University of Pretoria, Pretoria, South Africa

**Keywords:** South Africa, Climate change, Adaptation, Health systems, Heat, Extreme weather, Health policy

## Abstract

**Background:**

Globally, the response to climate change is gradually gaining momentum as the impacts of climate change unfold. In South Africa, it is increasingly apparent that delays in responding to climate change over the past decades have jeopardized human life and livelihoods. While slow progress with mitigation, especially in the energy sector, has garnered much attention, focus is now shifting to developing plans and systems to adapt to the impacts of climate change.

**Methods:**

We applied systematic review methods to assess progress with climate change adaptation in the health sector in South Africa. This case study provides useful lessons which could be applied in other countries in the African region, or globally. We reviewed the literature indexed in PubMed and Web of Science, together with relevant grey literature. We included articles describing adaptation interventions to reduce the impact of climate change on health in South Africa. All study designs were eligible. Data from included articles and grey literature were summed thematically.

**Results:**

Of the 820 publications screened, 21 were included, together with an additional xx papers. Very few studies presented findings of an intervention or used high-quality research designs. Several policy frameworks for climate change have been developed at national and local government levels. These, however, pay little attention to health concerns and the specific needs of vulnerable groups. Systems for forecasting extreme weather, and tracking malaria and other infections appear well established. Yet, there is little evidence about the country’s preparedness for extreme weather events, or the ability of the already strained health system to respond to these events. Seemingly, few adaptation measures have taken place in occupational and other settings. To date, little attention has been given to climate change in training curricula for health workers.

**Conclusions:**

Overall, the volume and quality of research is disappointing, and disproportionate to the threat posed by climate change in South Africa. This is surprising given that the requisite expertise for policy advocacy, identifying effective interventions and implementing systems-based approaches rests within the health sector. More effective use of data, a traditional strength of health professionals, could support adaptation and promote accountability of the state. With increased health-sector leadership, climate change could be reframed as predominately a health issue, one necessitating an urgent, adequately-resourced response. Such a shift in South Africa, but also beyond the country, may play a key role in accelerating climate change adaptation and mitigation.

## Background

The impacts of global changes in climate are rapidly escalating in South Africa. Unless concerted action is taken to reduce greenhouse gas emissions, temperatures may rise by more than 4 °C over the southern African interior by 2100, and by more than 6 °C over the western, central and northern parts of South Africa [1, 2]. Extreme weather events are the most noticeable effects to date, especially the drought in the Western Cape and wildfires, but rises in vector- and waterborne diseases are also gaining prominence. Global warming, which manifests as climate variability, has already been implicated in increased transmission of malaria, Rift Valley Fever, schistosomiasis, cholera and other diarrheal pathogens, and Avian influenza in the country [3–10]. Studies have documented the considerable impact of high ambient temperatures on mortality in the country, with mortality rises of 0.9% per 1 °C above certain thresholds, and considerably higher levels in the elderly and young children [11, 12]. Food security is under threat, with, for example, crop yields likely to decline in several provinces, with concomitant loss of livestock [13]. Moreover, any negative impacts of climate change on the country’s economy will have major implications for people’s access to food, which is largely contingent on affordability. Food access is already tenuous given the existing levels of poverty and as ownership of arable land is highly inequitable, reflecting the particular history of the country [14].

The impact of rises in temperature are especially marked in occupational settings, particularly in the mining, agriculture and outdoor service sectors [15–17]. Impacts, including measurable mortality effects, are heightened in those living in informal settlements, where houses are often constructed of sheets of corrugated iron [18–20]. In addition, heat increments are pronounced in many schools and health facilities as these have not been constructed to withstand current and future temperature levels [21, 22]. Importantly, all the impacts of climate change affect mental health, in a nation where already one sixth of the population have a mental health disorder [23].

While climate mitigation efforts, especially a reduction in carbon-based power production, have garnered much attention, focus is shifting to more direct, and shorter or ‘near’ term actions to counter the impacts of climate change [24–26]. These actions – commonly called adaptation measures – range from building the resilience of the population and health system, to preparing for health impacts of extreme weather events and to reducing the effects of incremental rises in heat in the workplace and other settings [27].

Most importantly, the effectiveness of adaptation pivots on reducing levels of poverty and inequities, especially in women and other vulnerable groups. Simply put: if an individual’s or household’s socio-economic status is robust, they will have a greater ability to withstand shocks induced by climate change. In South Africa, however, about a quarter of the population are unemployed and over half live below the poverty line [28]. Poverty reduction initiatives, such as the highly successful social grants system [29], thus lie at the heart of health adaptation. These initiatives already reach 17.5 million vulnerable people in South Africa [30], could be further extended to counter balance the disproportionate effects of climate change on vulnerable groups [31]. Equally, having a resilient health system is central to effective climate change adaptation.

While health professionals can play a critical role in advocating for stronger mitigation efforts such as a shift from brown to green energy (the government envisages that in 2030, still two thirds of energy production in the country will be coal-based [32]), the contribution of the health sector mostly centres around climate change adaptation. Several features of an effective health-sector adaptation response bear mention [33]. Firstly, national- and local-level policy frameworks and plans are required, supported by adequate resources. In particular, emergency incident response plans are needed for events such as heat waves, wildfires, floods, extreme water scarcity and infectious disease outbreaks [34]. These response plans set out the procedures to follow in the case of such events and the responsibilities of different actors. Secondly, communication is a key component of adaptation strategies, targeting a wide range of audiences, and using social and other media. Long-term communications strategies, such as “Heat education” campaigns, can raise awareness of the health risks of heat waves, and help prepare individuals and communities to self-manage their responses to increased heat [35]. Then, more short-term response communication is needed when an actual extreme weather event is forecast, making the public aware of an impending period of risk and what steps are needed to ameliorate that risk. Thirdly, the effectiveness of adaptation interventions rests on the strength of data systems and surveillance. Aside from providing warnings of extreme weather events, heightened surveillance is required of diseases associated with environmental factors, together with concerted efforts to systematically document the effectiveness of adaptation responses and to identify opportunities for improving services.

There is clearly a real opportunity to bring the credible voice and considerable resources of the health sector to bear on climate change policies and programmes [36–38]. It is important to assess the extent to which this is occurring and gaps in this response. Some reviews have examined this issue in South Africa [39–41], but none have done so recently, or employed systematic review methodology. This study fills that gap and presents lessons from the response in South Africa that might be applied in other countries and, indeed, globally [42]. In recent decades, South Africa has played a leading role in tackling public health issues affecting the African region, especially in the HIV field. The country has the potential, drawing on its research and programme expertise, to play a similar role in climate change adaptation, galvanising action in other parts of the continent. Thus, while the impacts of climate are somewhat unique to each country and even within different parts of a country, lessons drawn from this case study may provide useful insights for other countries in the region.

The paper is divided into two thematic areas. The first covers policy frameworks relating to climate change adaptation, as well as data monitoring and surveillance of climate change adaptation in the country. The second reviews the level of preparedness and actions already taken for extreme weather events, rises in temperature and infectious disease outbreaks. Topics indirectly related to health, such as food security, are not addressed in the paper, though remain of key importance.

## Review methods

We systematically reviewed literature indexed in PubMed (Medline) and Web of Science for articles that address climate change adaptation in South Africa. Full details and the PRISMA Flow Chart are described elsewhere [43]. The Pubmed search strategy included free text terms and controlled vocabulary terms (MeSH codes), specifically: (((((“South Africa”[MeSH]) OR (“South Africa”[Title/Abstract]) OR (“Southern Africa*”[Title/Abstract]))) AND “last 10 years”[PDat])) AND (((“global warming”[Title/Abstract] OR “global warming”[MeSH] OR climatic*[Title/Abstract] OR “climate change”[Title/Abstract] OR “climate change”[MeSH] OR “Desert Climate”[MeSH] OR “El Nino-Southern Oscillation”[MeSH] OR Microclimate[MeSH] OR “Tropical Climate”[MeSH])). This strategy was translated into a Web of Science search.

**Fig. 1:**
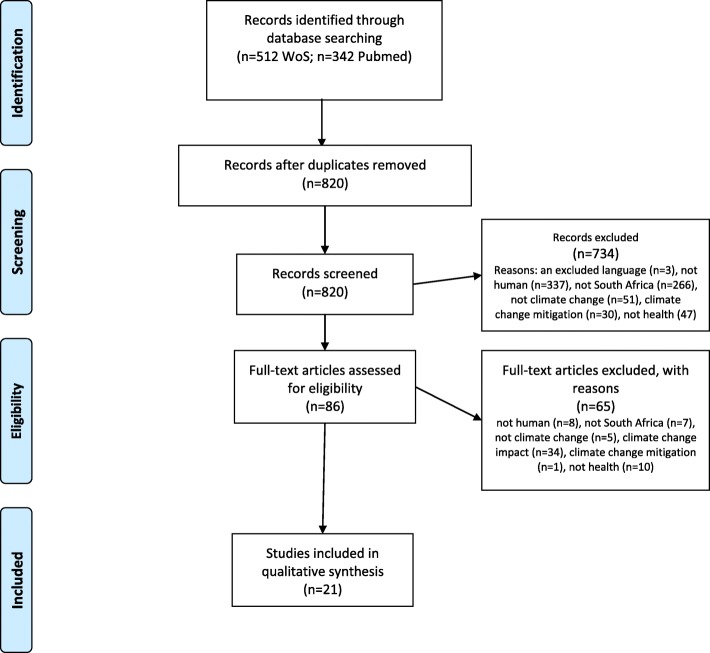
PRISMA Flow Diagram for Review of health-related adaptation to Climate Change in South Africa

In total, 820 titles and abstracts were screened by a single reviewer after removal of 34 duplicate items. To be included, articles had to describe adaptation interventions to reduce the impact of climate change on health in South Africa. All study designs were eligible and no time limits were imposed. We excluded articles that were not in English (*n* = 3), only covered animals or plants (*n* = 345), were not on South Africa (*n* = 273), were unrelated to health (*n* = 57) or to climate change (*n* = 56), or were only on climate change impact (*n* = 34) or mitigation (*n* = 31). In total, we screened 86 full text articles for eligibility, 21 of which were included (Fig. 1). We also included literature located through searches of article references (one additional paper) or through targeted internet searches. Thereafter, we extracted data on the characteristics of the included articles, including their study design and outcome measures (Table 1). In analysis, we grouped studies on similar topics and, where possible, attempted to highlight commonalities or differences between the study findings. Policy documents were located by searching the website of the National Department of Environmental Affairs (https://www.environment.gov.za) and the National Department of Health (http://www.health.gov.za/), and by asking experts familiar with these policies in South Africa.

**Table 1:** Characteristics of studies included in the review

Article title	Country	Study setting	Study design	Data sources	Analysis methods	Study population	Study aim	Theme	Study intervention	Outcome data	Outcome measures
Climate change impacts on working people (the HOTHAPS initiative): findings of the South African pilot study [17]	South Africa	Johannesburg, Gauteng Province and Kimberley, Northern Province	Mixed methods	Focus group discussions, in depth interviews and quantitative data on weather	Grounded theory	Outdoor workers (e.g. grave diggers, street sweepers, roadside construction workers and horticultural workers), supervisors and farm managers	Examine perceptions of workers about working in hot sun-exposed weather and to identify adaptation measures weather	Heat adaptation actions	Adaptation measures in occupational setting	Characteristics of adaptation response	Actions to protect outdoor workers from heat-related conditions
Information and communication technology and climate change adaptation: Evidence from selected mining companies in South Africa [72]	South Africa	Whole country	Effectiveness evaluation	Documents	Systematic literature appraisal	Mining houses	To determine measures undertaken by mining houses to use information, communication and technology (ICT) to address CC adaptation at different stages of the mining value chain	Health promotion	Use of ICT for: early warning systems for flooding, sharing knowledge of adaptation among staff, coordinating disaster recovery, supporting development of adaptation policies, analysing information for vulnerability assessments	Level of preparedness for CC-induced disasters, warning information	ICT changes in communication patterns for reducing CC vulnerability and exposure
Mind the gap: institutional considerations for gender-inclusive climate change policy in sub-Saharan Africa [31]	All of sub-Saharan Africa	Whole country	Narrative review	Documents	Feminist analysis	General population, vulnerable groups of women	To elucidate why women should be placed at the heart of CC interventions and establish connections between gender and CC	Health promotion	Institutions: the “sets of rules, decision-making procedures, and programs that define social practices, assign roles to the participants in these practices, and guide interactions among the occupants of individual roles”	Outcomes of policies or institutions	Performance of institutions with regard to gender
Seasonally lagged effects of climatic factors on malaria incidence in South Africa [55]	South Africa	Limpopo	Spatial and temporal mapping	Notified cases of malaria; Meteorological data (Gridded time-series climate data)	Spatial analysis	Adults and children with malaria	To analyse relationships between local climatic effects and remote atmospheric teleconnections on malaria incidence, including lag effects	Warning preparedness, surveillance using health and climate data	Document associations between malaria incidence and spatio-temporal climate variations	Infectious disease incidence	Effect of local and regional climate factors and large scale climate phenomena, on malaria incidence. Ability to predict timing of malaria incidence and inform early warning information
Climate change and occupational health: A South African perspective [15]	South Africa	Whole country	Narrative review	Document review	Not stated	People in the workplace	To review impacts of CC on occupational health and prevention and control measures	Health promotion	Prevention and control of heat in the workplace	Occupational health	NA
Long-run relative importance of temperature as the main driver to malaria transmission in Limpopo Province, South Africa: a simple econometric approach [3]	South Africa	Limpopo Province	Econometrics	South African Weather Services; European Centre for Medium-Range Weather Forecasts; Malaria Control Centre, Limpopo Province and Department of Health	Time series analysis	People with malaria	To examine the distribution of malaria, determine direction and strength of the relationship and causality between malaria and meteorological variables	Early warning system	Detect the time and length of impact on malaria cases using metrological variables	Infectious disease incidence	Malaria correlation with temperature and rainfall, and timing of cases
Inclusion of climate change strategies in municipal Integrated Development Plans: A case from seven municipalities in Limpopo Province, South Africa [46]	South Africa	Limpopo Province	Policy analysis	Documents	In depth content analysis	Residents in a municipality	Investigate the extent to which CC adaptation and mitigation strategies are embedded in: Integrated Development Plans (IDPs) (planning document for each local municipality, contains policies and frameworks)	Integration of CC into other policies and plans	Inclusion of CC into IDPs	Quality of CC policies	Presence in policy documents of proposals on how to adapt and mitigate against CC; report of awareness of level of vulnerability; degree to which policies have a structured approach
University students as recipients of and contributors to information on climate change: insights from South Africa and implications for well-being [62]	South Africa	Universities in Eastern Cape, KwaZulu Natal and Western Cape provinces. Some research activities in other provinces	Qualitative study	Focus group discussions, semi-structured interviews, key informant interviews and participant observation	Critical interpretivist research approach	University students	To explore influences of communication on CC on students’ understanding, concern and response; to explore barriers and opportunities for students when they inform their communities on CC; to recommend how to empower students as recipients of and contributors to information on CC	CC communication	Student’s receipt of communication on CC information and their communication of CC to others	Quality of CC communication	Perceptions of CC, role of new technologies in CC knowledge and communication; student leadership, nature of communication, CC knowledge and communication skills
National policy response to climate change in South Africa [42]	South Africa	Whole country	Narrative review	Documents	None stated	Whole population of the country	Analyse the national government’s plan on CC adaptation and mitigation, including for the health sector	Health policy and health systems	Plans for: adaptation measures for socioeconomic and environmental resilience and emergency response; activities for the health sector	Status of CC policy	NA
Health aspects of climate change in cities with Mediterranean climate, and local adaptation plans [47]	Australia, Chile, Spain, South Africa, United States	Cape Town, Western Cape Province	Narrative review	Climate action plans and other documents	None stated	Residents of Cape Town	To highlight health impacts of CC in Med-cities, analyze local climate adaptation plans and make adaptation policy recommendations	Policies and plans for CC adaptation	Adaptation plans that prevent or reduce vulnerability, including improvement of housing, infrastructure and the adaptation capacity of the population	Status of CC policy	Whether CC-related drivers of health impacts and key policy aspects were identified as risks, and what types of policy tools address the drivers
Re-imagining the potential of effective drought responses in South Africa [75]	South Africa	Drought-affected areas	Effectiveness evaluation	Key informant interviews, analysis of historical documents, academic literature and social media	Mixed methods approach	Government officials, academics, civil society and others working on drought response in South Africa	To assess the responses to droughts over time, and make recommendations for developing inclusive knowledge generation processes in future	Processes of adaptation response	Response to droughts	Characteristics of adaptation response	Characteristics of response of policy makers, scientists and others to droughts; extent to which these responses changed over time
Re-making the global economy of knowledge: do new fields of research change the structure of North–South relations? [100]	Australia, Brazil, South Africa	Whole country	Case series	Semi-structured key informant interviews	None stated	Researchers in CC, HIV and gender studies	To examine how global-North predominance in the making of organized knowledge has been affected by the rise of new domains of research	Knowledge generation for CC	Knowledge-making and knowledge circulation on CC	Characteristics of knowledge production	Assessment of equality in research resources, research structures, barriers to and tensions in research between global and Southern countries
Moving from adaptive to transformative capacity: building foundations for inclusive, thriving, and regenerative urban settlements [84]	South Africa	Bergriver Municipality, Western Cape Province	Case study	Qualitative interviews, dairy keeping	Mixed methods approach	Youth, community	To apply explore how transformative capacity can be built in practice	Building resilience	Work with unemployed urban youth, and introduce a ‘community currency’ into the informal business sector. Increase interaction between the municipality and youth	Understand resource flows and networks for adaptation response	Effects of transformative capacity
What role for local organisations in climate change adaptation? Insights from South Africa [49]	South Africa	Namaqualand, Northern Cape Province	Formative research	Survey of NGOs, CBOs and government officials	Mixed methods approach	Government officials, NGOs and CBOs	To develop and apply a framework to measure adaptive capacity among local organisations	Implementation of adaptation plan	Development and implementation of CC adaptation projects	Performance of adaptation response	Effectiveness and flexibility of local organisations and awareness of adaptation
Variations in approaches to urban climate adaptation: experiences and experimentation from the global South [101]	Ecuador, India, South Africa	Durban, KwaZulu Natal Province	Qualitative methods	Semi-structured key informant interviews	Thematic analysis	Government officials, local municipal and city officials	To examine climate adaptation planning approaches in three cities and analyse different planning pathways and forms of stakeholder involvement	Integration of CC into other policies and plans	Prepare for CC through reducing vulnerability and enhancing resilience of populations, assets and municipal operation	Performance of adaptation response	Potential of different adaptation approaches and implications for government coordination, and participation and adaptive capacity of vulnerable groups
What enables local governments to mainstream climate change adaptation? Lessons learned from two municipal case studies in the Western Cape, South Africa [44]	South Africa	Western Cape Province	Case studies	Semi-structured key informant interviews; documents	Thematic analysis	Residents of local areas	To identify factors that enable action to be taken at local government level	Integration of CC into other policies or plans	CC adaptation plan	Performance of adaptation response	Role of dedicated environmental champions in political leadership. Costs of CC
A normative model for integrating organisations for disaster risk reduction and climate change adaptation within SADC member states [69]	Botswana, Madagascar, Malawi, Namibia, South Africa, Swaziland, Tanzania, Zambia and Zimbabwe	Gauteng	Mixed methods	Documents, interviews, online survey	Thematic analysis and triangulation	Government officials	To investigate the actors and their location in government that create and shape governance in disaster risk reduction and CC adaptation integration	Integration of CC into other policies or plans	Disaster risk reduction and CC adaptation	Level of preparedness for CC-induced disasters, warning information	Level of integration of government organisations
Local climate governance in the Global South: the case of eThekwini Municipality and the Responsible Accommodation Campaign [51]	South Africa	eThekwini, KwaZulu Natal Province	Case study	Documents, observation of meetings and forums, key informant interviews, quantitative and qualitative questionnaires	Action research project with mixed methods for data analysis	Government officials, local municipal and city officials	To identify different forms of local climate governance with a specific focus on relevance of networks	Importance of network governance approach to impact climate governance	Network governance	Outcomes of policies or institutions	Ability of network governance to shape climate policy and alter a project and its implementation
Climate change and vulnerability discourse by students at a South African university [63]	South Africa	Limpopo Province	Survey	Self-report survey questionnaire	Univariate and bivariate analysis	University students	To assess the knowledge and understanding of CC and its impacts by university students by faculty and gender	CC knowledge	University curricula	Awareness	Level of knowledge and understanding of CC
Climate change impacts and adaptation in South Africa [40]	South Africa	Whole country	Narrative review	Publications and grey documents	Not stated	General population	To review current approaches and recent advances in research on climate impacts and adaptation	Integration of CC policy in other policies; status of adaption	Adaptation efforts in the country	NA	A wide range of adaptation measures
Contesting adaptation synergies: political realities in reconciling climate change adaptation with urban development in Johannesburg, South Africa [71]	South Africa	Johannesburg, Gauteng Province	Case study, qualitative methods	Key informant interviews, documents	Not stated	Urban planning actors, including political decision makers, policy-makers and executive leaders	To investigate contextual factors that shape adaptation barriers, and to explore how adaptation practices can be implemented through synergistic responses, in infrastructure and land use planning practices Flooding used as illustrative example	Urban health	Use of CC adaptation synergies in planning practices	Performance and characteristics of adaptation response	Synergies between planning responses to flooding and urban planning. Government planning priorities. Communities perceptions

CC climate change

## Results

### Engagement of the health sector in climate change policies, planning and data systems

We located 14 journal articles on health sector engagement. With these limited number of records, results are presented as a narrative, rather than as a comparison of findings in different parts of the country or across population groups. We first discuss national and local policies and practices, and then turn to assess the climate and health monitoring systems in the country.

In recent years, the national government has developed a series of documents covering key legislative and strategic aspects of adaptation. In 2018, the government released a draft of the National Climate Change Response White Paper which sets out the different ways in which climate change considerations can be integrated within all sectors, including health. This document updates the 2011 White Paper on this topic. More recently, the draft National Climate Change Bill was made available for comment [24]. Little reference is made to human health and scanty detail is provided on actual implementation of the policies. Additionally, in 2017, the second draft of the South African National Adaptation Strategy was made open for public comment [25]. This is a ten-year plan, which describes key strategic areas, with measurable outcomes. The strategy acts as a reference point for all climate change adaptation efforts in South Africa, providing overarching guidance across the various sectors of the economy. As such, it seeks to ensure that different levels of government and the private sector integrate and reflect climate change adaptation. The implementation priorities for health are listed as water and sanitation, early warning systems for effective public health interventions during extreme weather events, and occupational health.

While national policies set the stage for lower levels of government and funding prioritisation, much of the actual planning for climate change adaptation occurs at the provincial and local government level. Most importantly, each local area government is charged with developing an Integrated Development Plan every five years, involving many sectors, including health [44]. Health implications of climate change are mentioned in some of these plans, but not all [45–47]. A survey of Environmental Health Practitioners (*n* = 48), who are at the forefront of implementing these plans, provides insights of the degree to which climate change priorities have been incorporated within these plans [48]. Though almost all felt that they should play a supportive or leading role in addressing climate change, only half had a budget allocated for climate change and health-related work, and only a third had ever participated in climate change-related projects. Another study involving fieldwork in a range of settings in South Africa reported that, for climate change adaptation plans to be successful, local communities need to be more involved in their design and implementation [49]. A further study in eThekwini Municipality, KwaZulu-Natal Province noted that few climate change advocates had emerged among local politicians and civil servants, and that decisions made at the local government level seldom took climate change issues into account [50]. A case study of the Integrated Development Plan in the same municipality examined the working relations between the local government, civil society and private sector actors on climate change initiatives, forming a ‘network governance’ structure [51]. Having a ‘network’ helped local government shift from ruling by regulations and authority, to a ‘softer approach’, one that ‘enabled’ solutions to climate challenges. For their part, however, the private sector found it challenging to incorporate climate-sensitive actions into their *modus operandi* and may require financial incentives to adopt mitigation and adaptation measures. Concerns remain that the private sector - and indeed the public sector – view environmental issues as constraints to profit and development, rather than as contributors [50].

While it appears that national and local policy and planning frameworks can influence programmes and funding allocations, at least to some extent, their impact needs to be monitored closely, using appropriate indicators. These data can help decision-makers to identify programmatic areas to target, researchers to analyse and benchmark programme performance, and civil society and communities to gauge service provision in their area. The growing and shifting burden of climate-sensitive diseases, however, means that the district- and national-level indicators currently used for monitoring disease and service provision may be less relevant in this new era.

A review in 2014 emphasized the need for developing new tools for incorporating data from climate monitoring systems, for example temperature and rainfall, into Demographic Health Information Systems (DHIS) in South Africa, and vice versa [39]. The tremendous potential of integrated weather-health data is, however, constrained by differences in spatial, temporal and quality of these respective data sources. While weather data are recorded hourly and in small geographical units, [52, 53] health data are often only available in monthly units and at district level. Analysing climate data at those resolutions results in a considerable loss of information and thus predictive ability. Challenges in collecting health data – often paper-based – means that these data are often of poorer quality than climate data, though deficiencies in climate data are not uncommon in South Africa [12]. Despite these limitations, combining climate and health data can assist with seasonal forecasting, and early warning systems for infectious diseases and other climate-related conditions.

The Infectious Diseases Early Warning System project (iDEWS) project, involving Southern African and Japanese researchers, aims to advance all these efforts, and to develop early warning system for a wide range of infectious diseases, based on climate predictions [54]. Such applications have been developed to support malaria programming in the country [55], where temporal patterns in temperature, rainfall and sea surface temperature can forecast changes in malaria incidence and the geographical expansion of disease outbreaks [3, 56, 57]. Further, as shown in a study in Cape Town, close monitoring of ambient temperature, can predict spikes in incidence of diarrhoeal disease, allowing health services to prepare for rises in admissions and outpatient visits [9]. Similarly, another study across several provinces noted that anomalous high rainfall precedes outbreaks of Rift Valley fever by one month and that this finding can be used to forewarn epidemics in affected areas of the country [58].

In addition to applications around infectious diseases, health and climate data are analysed in multiple-risk systems, such as the South African Risk and Vulnerability Atlas (SARVA) [59]. This spatial database allow for visualisation of the drivers, exposures, vulnerabilities, risks and hazards across different locations. SARVA provides more than just data outputs, however, and has developed a range of practical climate services for the agriculture sector, for example. Additionally, Heat–Health Warning Systems in the country, based on increasingly sophisticated meteorological systems, have long lead-times, and can alert decision-makers and the public of forthcoming extreme heat events, triggering a graded set of pre-specified actions [52, 60].

While adaptation is classically defined as the ability to deal with change, it also encompasses the capacity to learn from it. Doing so requires investments in research and analytical systems, especially among public health practitioners. Of concern, a collaboration across several countries, including South Africa, noted that climate change and environmental health, in general, have not been mainstreamed within curricula at medical schools [61]. The group noted that, given the limited capacity in this area, international assistance maybe required to develop curricula and teaching materials. Other studies in have documented considerable gaps in knowledge on climate change among university students across disciplines and the limited ability of these future leaders to engage with others on the topic [62, 63]. Overall, the research outputs by South Africa scientists on climate change has grown (around 600 academic publications in 2015), but only 3%, or about 20, of these publications make reference to health [64]. Of more concern, a report of the Lancet Countdown on health and climate change group, using a narrower search strategy, located only about 20 papers related to climate change and health in the whole of Africa in 2017, constituting well under 10% of the total 300 such papers worldwide [65]. Reviews have also noted that little interdisciplinary work between meteorology and health has been done [66]. But, perhaps most importantly, research investigating the performance of interventions to reduce the health impacts of climate change are largely absent [40, 67].

### Response to extreme weather events and gradual increments in temperature

We located only 8 studies applicable to this section of the review, limiting our ability to provide a comprehensive analysis on the topic at hand. This section covers disaster preparedness and responses, including of the health system, and the population groups, occupations and housing types most vulnerable to heat exposure.

The government of South Africa has developed Disaster Management Frameworks and a National Disaster Management Centre, [25, 68] whose responsibilities include directing the country’s responses to disasters and strengthening cooperation amongst different stakeholders. There are, however, concerns that disaster risk reduction systems operate in isolation from other climate change adaptation initiatives in the country, rather than drawing on the strengths of each group [69]. While there are robust ‘Heat Health’ warning systems in the country, it appears that actual action plans or responses to heat waves require further development [35, 70]. Some steps have been taken to develop these systems in local government areas and the private sector. A case study examining preparedness for flooding in the city of Johannesburg provides useful examples of potential synergies between the health and other sectors, but also notes considerable political barriers to cross-sectoral actions [71]. Another example of preparedness was noted in a report by a mining company that operates in several parts of the country. The company had developed substantial information, communication and technology capacity for risk assessments, and warning systems for flooding and other climate-related disasters [72].

Efforts to prepare the health system for extreme weather events or infectious disease outbreaks are hampered by weaknesses in health systems, especially in human resources for health in South Africa [28]. The recent experiences with the Listeriosis outbreak, the largest and longest lasting epidemic documented worldwide to date, brought these concerns to the fore, in particular the country’s ability to mount a swift and systematic response to disease outbreaks [73]. There were major challenges in collecting data on patient outcomes during the epidemic, for example, where the mortality status was unknown for as many as 30% of affected patients [74]. This outbreak and recent extreme weather events present many opportunities for learning. It seems, however, that these learning opportunities are often missed. A review of the responses to droughts in the country over the past century found that there have been few attempts to learn from previous droughts, and that responses to each event were largely developed de novo, rather than shaped by long-term planning and lessons from previous similar events [75].

Several populations groups and geographical areas in South Africa are especially vulnerable to the impacts of climate change. The Draft National Adaptation Strategy in 2017 and the White Paper of 2011, which presented the South African Government’s strategic vision for an effective climate change response mentions the importance of placing women and other vulnerable groups at the centre of adaptation actions. These documents, however, do not expand on this concept and no evidence was located on the differential effectiveness of adaptation interventions among women in the country, and efforts to specifically tailor adaptation measures accordingly [31]. This is concerning as many of the health and social burdens in the country are underscored by harmful gender norms, with, for example, the country has one of the highest rates of sexual violence worldwide and a very gendered HIV epidemic [76]. Few studies were located on adaption in occupational settings, many of which may become ‘moderate to high risk’ workplaces as temperatures rise [15]. A study in Johannesburg and Upington (where daily maximum temperatures may exceed 40 °C) found that outdoor workers experienced a range of heat-related effects [17]. These include sunburn, sleeplessness, irritability and exhaustion, leading to difficulty in maintaining work levels and output during very hot weather. Aside from commencing work earlier, during the cooler part of the day, no measures had been taken to protect the workers, who believed that sunglasses, wide-brimmed hats and easier access to drinking water would improve their comfort and productivity. In the mining sector in South Africa, several studies have reported that workers’ comfort and productivity can be raised with interventions such as ventilation cooling [77–79]. Of note, insulation within many hospital buildings has been found wanting, but little had been done to address the problem [80]. Some hospitals have taken steps to increase use of natural ventilation to adapt to temperature increases and as part of efforts to curb use of air conditioning [81]. Natural ventilation also reduces transmission of multi-drug-resistant tuberculosis, important as the country has one of the highest rates of tuberculosis worldwide [82].

Improvements in specific types of housing, especially in informal settlements, could reduce the considerable heat-health impacts of these structures, which include mortality [18, 19]. We identified several studies on urban health in South Africa, but these did not extend to documenting the health benefits of energy efficient buildings, green spaces, public transport, car-free zones and active transport [71, 83, 84]. Further, many school classrooms in the country are constructed of prefabricated asbestos sheeting and corrugated iron roofs or made from converted shipping containers. A study in several parts of Johannesburg showed that heat-related symptoms are common in these structures [21]. The authors postulate that improving these structures would increase comfort for scholars and could raise educational outcomes.

## Discussion

The review sums the body of evidence on climate change adaptation in South Africa. We note that some steps have been taken to develop a multi-pronged strategy that cuts across health and other disciplines, and that helps adapt to the already substantial and future impacts of climate change in the country [42, 85]. Such steps are being supported by efforts to build the resilience of vulnerable groups, who have limited ability to adapt to droughts, flooding, changes in biomes and other events [84]. While key policy frameworks are in place, it is difficult to gauge whether these have been actualized at national and local level. Increased efforts to include civil society advocates, local communities and the private sector may accelerate progress with policy implementation. In South Africa, highly-detailed data are available on weather conditions at very fine spatial and temporal resolution. Health data generally have lower resolution and quality. Additional spatial and temporal disaggregation of health information could provide invaluable data, for example, to help identify critical heat-stress thresholds in different settings and to monitor the effectiveness of action response plans. In the meantime, more evaluations, including ‘dry runs’ are needed of the health aspects of emergency response plans to extreme weather events [60]. Gaps were also noted in research infrastructure and in efforts to reduce heat exposures in some housing types and occupational settings.

The case study presented here provides useful perspectives for other countries in sub-Saharan Africa. Most especially, the findings could feed into the work of the Clim-HEALTH Africa network, which aims to share expertise, and to inform climate-sensitive policies and planning across the region [86]. While the network has already supported the development of several adaptation plans, the evidence presented here may contribute to future iterations of these plans and other network initiatives.

Strategies for extreme events – and indeed for all interventions related to climate change – need to be informed by an analysis of the implications for those living in poverty, migrants, women and children, among other groups. We noted little evidence of specific ‘targeting’ of adaptation responses to vulnerable groups. There may, for example, be benefits to specifically targeting women, as opposed to men, in early warning systems and disaster reduction plans. This approach is supported by evidence that, as with many other social interventions, it is most effective to distribute relief kits and house building grants to women [87]. In tandem with other adaptation initiatives and targeting, the overall functioning of the health system needs to be fortified, though there is much uncertainty about how this might be done [88, 89]. The goal is to ensure that health facilities remain operational during extreme weather events, serve as places of refuge and support, and can summon the additional capacity required to deal with the impacts of extreme events. An external evaluation of the recent response to the Listeriosis outbreak might identify important lessons for improving the response to future outbreaks or extreme weather events. Potential links between climate change and that outbreak as well as future outbreaks also warrant investigation [73]. The health sector is also responsible for developing and testing heat-health guidelines for specific settings and populations, such as guidelines for sports events, which stipulate the temperature thresholds at which different sport activities should be cancelled.

Going forward, there are many opportunities to strengthen data monitoring and surveillance systems on climate and health. The Lancet Countdown has developed indicators to monitor national-level progress on climate change in the health sector [90]. Six of these pertain to adaptation and correspond broadly to the sections of this paper: 1. National adaptation plans for health; 2. City-level climate change risk assessments; 3. Detection and early warning of, preparedness for, and response to health emergencies; 4. Climate information services for health; 5. National assessment of vulnerability, impacts and adaptation for health; and 6. Climate-resilient health infrastructure. This paper suggests that additional work is required in each of these areas in South Africa. These indicators – and the full Lancet Countdown framework – could be used to benchmark the country’s progress against other nations and to pinpoint the specific areas requiring attention [91]. Monitoring data could be used to produce annual estimates of the burden of disease and health costs that would be averted by more vigorous climate change mitigation or adaptation efforts [92]. Such disease prediction models have been used with great effect in the HIV epidemic [93], where they generated considerable pressure on the government and international donors to prioritise actions and resource allocations accordingly. Additionally, given the vulnerabilities of food security to climate change in South Africa, close monitoring is needed of under-nutrition, agriculture and marine productivity [14, 94].

An adequate adaptation response is contingent on the progressive accrual of robust evidence. This, in turn, depends on earmarked funding for research on climate change and health, agile and responsive research systems and, indeed, an adequate number of capacitated researchers. Given the growing attention paid to this field, high-quality evidence with compelling findings could rapidly foment policy changes. Moreover, if the quality and volume of research were raised, it will become possible to make evidence-based national policies, as in other health fields. The health sector in South Africa, with its considerable research capacity, is well placed to lead such efforts. To achieve this, however, researchers in other health fields, such as HIV, for example, would need to take on projects on climate change. As a first step, it may be useful to convene consultations of experts in health, the environment and related fields to develop broad plans for taking advantage of opportunities for cross-learning and action. Some targeted research funding for joint health and environmental projects on climate change could have a considerable impact. The iDEWS project offers an important example of such an initiative [54]. In the long run, research in this field could be sustained by allocating more time to climate change topics in training programmes for health workers and public health practitioners.

While the review highlights some important findings, the limited number of papers located suggests that the country has some way to go to fulfilling its potential leadership role on the continent, and indeed globally. One area that health practitioners in South Africa could lead on is the promotion of a ‘meat tax’, given their pioneering work on the ‘sugar tax’ [95]. Curbing the intake of ruminant meat is a key climate change mitigation strategy and would lower cancer risks, among other health benefits [96]. This is important in South Africa, where an estimated total of 875,000 tons of beef are consumed annually [97], producing 648 gigagrams of methane [98]. The principal arguments for a sugar tax – and indeed for tobacco and alcohol taxes – hold for ruminant meat: harm to self and others, and the considerable cost burdens on broader society [99]. In this case, the harms are mediated through environmental destruction, a change in climate and cancer, amongst others [95]. Such policies are, however, likely to be vigorously opposed by the meat industry in South Africa, and public health and environmental and social justice experts in the country will need to rally together [26]. Bringing together the complementary skills of these experts has the potential for powerful synergies and for drawing additional researchers into the climate change and health arena. Similarly, broadening the scope of climate change adaptation to encompass existing programmes that have an indirect impact on climate change adaptation would also increase the number of climate adaption workers. This would also assist in mainstreaming climate change into existing health programmes, and highlight additional ways that the health sector has successfully responded to the problem. Increased attention to these successes might demonstrate the extent to which the sector is leading the field and its potential contribution to overall adaptation efforts in the country.

The study has some limitations. The limited number of papers included in the review (*n* = 22) and the heterogeneous nature of the evidence constrained our ability to draw overall conclusions about the adaption response to date. Likely many additional studies on the topic are published in grey literature sources or unpublished and would thus not be in our search. Moreover, the search would not have located studies of interventions by the health sector that indirectly reduce the impact of climate change, but have not been framed as such. These intervention may include socio-economic initiatives that build financial resilience of households, improvements in housing and control of infectious diseases.

## Conclusion

In fact, explicitly framing existing programmes that have an indirect impact on climate change adaptation as contributing to climate change adaptation.

The review highlights several important gaps in adaptation practices. While policy and planning frameworks for climate change at national, provincial and local level do make mention of health priorities, the health sector does not yet appear to be viewed as an essential platform for adaption measures, and health concerns appear to be accorded low priority. We did, however, note several important examples of health sector involvement in adaptation initiatives within local area government and in occupational settings. Importantly, there have been few rigorous evaluations of the effectiveness of actual interventions on climate adaptation for the health sector; most studies are descriptive in nature. Perhaps the largest knowledge gap is evidence around the effectiveness of disaster management systems and the level of preparedness of these systems for extreme weather events. The lack of studies on that and other topics may reflect the nascent nature of the field and that the priority given to climate-sensitive conditions in training for health workers and public health practitioners has not reflected the present and future burden of these conditions.

Clearly, interventions targeting the direct impacts of climate change need to occur in tandem with actions to shore up the resilience of the population and health system. Many health sector initiatives targeting those areas already contribute to climate adaptation, albeit indirectly so. Highlighting the successes of these initiatives and explicitly framing them as part of climate adaptation could mainstream climate change into existing programmes and provide examples of the ways in which the country is already successfully responding to the problem. Reframing in this manner may generate the leadership and momentum necessary for making rapid advances in this field.

Indeed, increased health sector leadership and lobbying may prove pivotal in advancing the adaptation field per se. The explicit framing of climate change adaptation and mitigation as critical to protecting the health of the nation may secure a more vigorous policy and programmatic response by government, and strengthen the engagement of civil society and communities [36]. Health could be placed firmly at the centre of policies for climate change adaptation and mitigation. Equally, effective leadership would mainstream climate change considerations into *all* policies for health [37]. High-quality research, involving a range of disciplines and backed by local and international funding, could go a long way to securing these changes.

While the country has led the way globally in HIV and several other arenas, it has yet to fully assume a leadership role in this field. With increased focus, the health sector could use its considerable influence to advocate for policy change and improved climate governance: it’s time for health to take a lead.

## Abbreviations

DHIS: Demographic Health Information System
HIV: Human Immunodeficiency Virus
iDEWS: Infectious Diseases Early Warning System project
